# Coldwater fish in a warm water world: Implications for predation of salmon smolts during estuary transit

**DOI:** 10.1002/ece3.7840

**Published:** 2021-06-28

**Authors:** Matthew L. Nobriga, Cyril J. Michel, Rachel C. Johnson, John D. Wikert

**Affiliations:** ^1^ U.S. Fish and Wildlife Service San Francisco Bay‐Delta Fish and Wildlife Office Sacramento CA USA; ^2^ National Marine Fisheries Service Southwest Fisheries Science Center Santa Cruz CA USA; ^3^ Center for Watershed Sciences National Marine Fisheries Service Southwest Fisheries Science Center Davis CA USA; ^4^ U.S. Fish and Wildlife Service Lodi Fish and Wildlife Office Lodi CA USA

**Keywords:** chinook salmon, largemouth bass, predation, predator–prey, striped bass, survival, warming climate

## Abstract

1. Predator–prey systems face intensifying pressure from human exploitation and a warming climate with implications for where and how natural resource management can successfully intervene. We hypothesized young salmon migrating to the Pacific Ocean face a seasonally intensifying predator gauntlet when warming water temperature intensifies a multiple predator effect (MPE) from Striped Bass *Morone saxatilis* and Largemouth Bass *Micropterus salmoides*. We evaluated this hypothesis using data synthesis and simulation modeling.
2. Contemporary studies based on acoustically tagged fish reaffirmed older observations that Chinook Salmon smolts must transit the Delta before water temperature reaches 20°C or mortality will be nearly 100%. Striped Bass attack rates on tethered smolts were insensitive to distance from shore and water temperature, whereas Largemouth Bass attack rates were highest near shorelines in warm water, supporting the temporal aspect of the hypothesis. Whether the combined effects of the two predators produce an MPE remains unconfirmed due to limitations on quantifying salmon behavior.
3. We used multiple simulation models to try to reconstruct the empirical relationship between smolt survival and water temperature. Simulations reinforced attack rate results, but could not recreate the temperature dependence in smolt survival except at higher than observed temperatures. We propose three hypotheses for why and recommend discerning among them should be a focus of research.
4. We found significant linear relationships between monthly mean inflow to the Delta from each of its two largest tributaries and monthly mean water temperatures along associated salmon migration routes, but these relationships can be nonlinear, with most of the correlation occurring at low inflows when water temperature is largely controlled by air temperature and day length. As the global climate warms, changed circumstances in predator–prey relationships may present important challenges when managing species vulnerable to extinction in addition to presently more abundant species.

## Graphical abstract

We build upon previous conceptual models of how intersecting impacts of multiple predators can render former habitats unsuitable to migratory animals and test the conceptual model with a case study. The case study focuses on the potential impacts of two non‐native predators on Chinook Salmon smolts as the young salmon migrate through California's Sacramento‐San Joaquin Delta on their way to the Pacific Ocean.

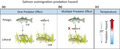

## 1 INTRODUCTION

By exploiting fish and wildlife, humans insert themselves into predator–prey systems in multiple ways. Most directly, we are predators of the organisms being exploited (Dairmont et al., 2015; Walters et al., 2005), but we also compete with other predators, change habitat mosaics, and introduce new species into ecosystems, all of which in turn affect predator–prey outcomes (Essl et al., 2011; Marshall et al., 2016; Mooney & Cleland, 2001; Roemer et al., 2001). Increasingly, we are changing the global climate which has multiple consequences including further alteration of predator–prey dynamics and limits to where and how individuals and organizations can respond with management interventions (Barton & Schmitz, 2009; Crozier et al., 2019; Draper & Weissburg, 2019).

Scientific evaluations of fish and wildlife management frequently emphasize understanding predator–prey systems (Gascoigne & Lipcius, 2004; Walters & Juanes, 1993). This is logical given the centrality of predator–prey systems to the ecosystem functions that ideally must be maintained to support exploited resources. Natural predator–prey systems can be challenging to mechanistically understand, particularly when multiple predators are involved because of nonadditive multiple predator effects (MPEs). By definition, an MPE occurs when *I* ≠ *P_1_* + *P_2_*, where *I* is the cumulative impact of two predators and *P_1_* and *P_2_* are the impacts of each predator in isolation. Researchers have reported MPEs from many systems involving both predator interference, *I* < *P_1_* + *P_2_* (Vance‐Chalcraft & Soluk, 2005), predator facilitation, *I* > *P_1_* + *P_2_* (Hixon & Carr, 1997), and combinations of the two (Soluk, 1993).

Scientific emphasis has led to rapidly increasing understanding of predator–prey systems (e.g., Ahrens et al., 2012; Verdolin, 2006), but has also increased our awareness of frequent context‐dependence between predators and prey (Lone et al., 2017; Northfield et al., 2017). For instance, Lone et al. (2017) showed that for most of the year a Norwegian population of roe deer (*Capreolus capreolus*) could, via diel changes in habitat use, balance its need for food and shelter against the risk of being killed by human hunters or lynx (*Lynx lynx*). During winter, however, the risk of starvation and freezing forced roe deer into an ecological “catch‐22” in which they were compelled to spend a greater amount of time in densely vegetated habitats efficiently hunted by lynx. Such spatiotemporally variable predation risk trade‐offs are likely the rule rather than the exception in nature (Verdolin, 2006). We note that predatory impacts constraining the success of conservation interventions on behalf of prey species could involve an MPE of the form *I* > *P_1_* + *P_2_*, but an MPE is not a necessary condition as an additive two predator threat (*I* = *P_1_* + *P_2_*) could still limit options for a prey species. Thus, context‐dependent predator–prey dynamics provide opportunities to evaluate local conformance or divergence from general patterns. This fuller understanding can in turn lead to better predictive models, which then better inform fish and wildlife management strategies (e.g., Gregory & Long, 2009).

**FIGURE 1:**
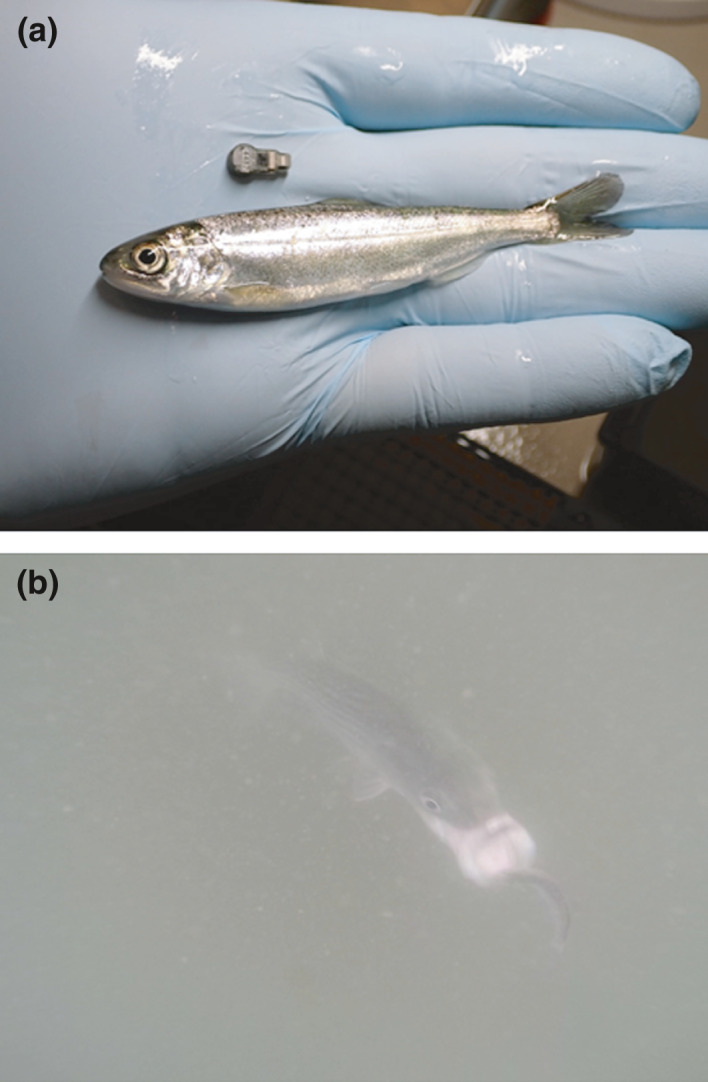
Upper: A Chinook Salmon smolt being prepared for surgical implanting of the acoustic tag immediately above it. Photo credit Arnold J. Ammann. Lower: Photograph of a Striped Bass attacking a Chinook Salmon smolt tethered to a Predation Event Recorder in California's San Joaquin River

Here we conceptualize and evaluate how a warming climate may be changing predator–prey outcomes for juvenile Chinook salmon (*Oncorhyncus tschawytscha*; Figure 1a) as they transit the San Francisco Estuary, California, USA. Salmon in the San Francisco Estuary are a model species and system since here Chinook Salmon are at the southern range of the species distribution and serve as an early indicator for how a warming environment can modify predator–prey interactions relevant to other taxa. The San Francisco Estuary is a highly modified ecosystem (Cloern & Jassby, 2012) and one of the most invaded estuaries in the world (Cohen & Carlton, 1998). The estuary's flow regime, habitat mosaics, food web, and fish communities bear little resemblance to what they did at the dawn of the California Gold Rush in 1849 when landscape conversion and water quality degradation began rapidly increasing (Brown et al., 2016; Gross et al., 2018; Nobriga & Smith, 2020). On their way to the Pacific Ocean, Chinook salmon smolts navigate past multiple predators in the San Francisco Estuary and its watershed, but we focus on the two most abundant piscine predators at the river‐estuary transition, Striped Bass (*Morone saxatilis*), and Largemouth Bass (*Micropterus salmoides*). Neither of these species is native to California, but both are abundant, popular sport fish with richer datasets than less common predators. We use the San Francisco Estuary as a case study because within it, salmon and their predators demonstrate an evolving interaction observed within a system that from a phenomenological perspective is data‐rich, but the mechanisms driving observed patterns are less well understood (Buchanan et al., 2018; Michel, Henderson, et al., 2020; Perry et al., 2010).

**FIGURE 2:**
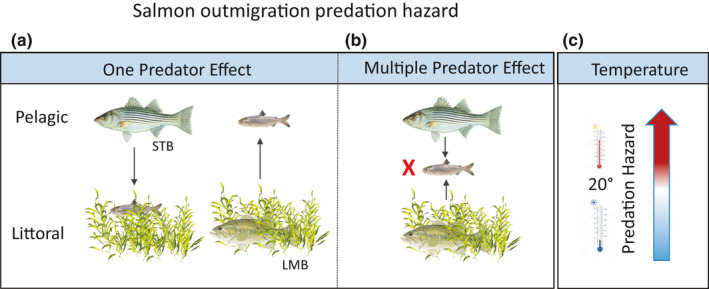
Conceptual model of a water temperature‐mediated multiple predator effect involving Striped Bass and Largemouth Bass preying on Chinook Salmon smolts as the latter migrate through California's Sacramento‐San Joaquin Delta on their way to the Pacific Ocean. When either Striped Bass or Largemouth Bass are present, salmon can use either littoral or pelagic habitats (a) but when both are present, salmon face increased predation hazard (b) during outmigration through a gauntlet of predators. Seasonal shift in temperatures above 20 degrees causes a nonlinear increase in predation hazard and intensifies the risk for salmon seeking refuge in littoral habitats

We hypothesize that Chinook Salmon smolts emigrating to the Pacific Ocean from California's Central Valley rivers face seasonal variation in the threat posed by multiple predators in a manner analogous to what was described above for roe deer in Norway (Lone et al., 2017). Rather than winter being the stressful season, it is the warming spring in California which eventually raises water temperature in the estuary to lethal levels (approximately 20°C or higher; Kjelson & Brandes, 1989). Striped Bass (Figure 1b) and Largemouth Bass are two important predators with divergent habitat use and hunting strategies, and as a result may restrict habitat options for migrating Chinook Salmon (Figure 2). Largemouth Bass metabolism is highly sensitive to the range of water temperatures that occur in the Delta during spring (see Results). This accelerates demand for prey as water temperatures warm, which in turn may limit migratory habitat options by rendering use of littoral habitats by smolts increasingly risky. We further hypothesize that the combined effect of these two predators manifests as an MPE that operates analogous to that reported by Hixon and Carr (1997). In Hixon and Carr’s (1997) example, coral reef damselfish are prey that can be vulnerable to pelagic jacks that attack from open‐water and reef‐associated groupers that attack from the reef itself. Comparably, for salmon in the Delta, Striped Bass roam the pelagic zone and Largemouth Bass are structure‐oriented ambush predators in the nearshore littoral habitat. When both predators are present, salmon exhibit high mortality as they confront the gauntlet of predators in the littoral and pelagic habitats as they migrate to sea (Figure 2). Using a combination of data synthesis and simulation modeling, we evaluated our conceptual model guided by three questions. (1) At what water temperature threshold will salmon not survive transit through the Delta, and how does that compare to indicators of predation risk? (2) Can we recreate empirical survival estimates with simulation modeling? (3) What are the implications for smolt survival of river flow and water temperature relationships in the Delta?

## 2 STUDY AREA

California's Central Valley is drained by the Sacramento and San Joaquin river systems, which flow into the San Francisco Estuary, a highly altered but well‐studied ecosystem (Cloern & Jassby, 2012). The Sacramento and San Joaquin basins support four runs of Chinook Salmon, differentiable by the timing of adult returns (Yoshiyama et al., 1998). Central Valley Chinook Salmon natural production has been greatly reduced due to extensive dam‐building and other ecological changes. Winter‐ and Spring‐run Chinook Salmon are listed under the U.S. Endangered Species Act; Fall‐ and Late‐fall run, though more numerous, have been largely overwhelmed by hatchery populations, the result of long‐term reliance on production hatcheries built to mitigate for large dams on major Central Valley river systems (Barnett‐Johnson et al., 2007; Sturrock et al., 2018). On their way to the Pacific Ocean, all Central Valley Chinook Salmon must emigrate through the Sacramento‐San Joaquin Delta, a network of channels at the eastern edge of the estuary (hereafter, Delta).

The survival of Chinook Salmon smolts transiting the Delta generally declines as a function of declining river flow and increasing water temperature, historically reaching nearly zero by the time waters warmed to 20°C (Kjelson & Brandes, 1989). It is within the physiological capacity of local salmon stocks to survive in water of 20°C (Marine & Cech, 2004), and from a bioenergetics perspective, it can be advantageous for them to do so if they have enough food (Sommer et al., 2001). Therefore, low survival at 20°C is widely believed to be caused by predation. Lines of evidence for this hypothesis include temperature‐dependent increases in predator attack rates (Michel, Smith, et al., 2020) and decline in maximum swim speeds that lessen the smolts’ quick‐burst escape response (Lehman et al., 2017). Striped Bass has been the estuary's dominant piscine predator for about 140 years (Nobriga & Smith, 2020). This facultatively anadromous, open‐water hunter can be distributed from tributary rivers to the Pacific Ocean. The ecological success of Striped Bass means that salmon smolts have likely been confronted with the constraints of this predator for many decades. Nonetheless, changing conditions can change the context within which Chinook Salmon and Striped Bass interact in a food web.

Largemouth Bass were also introduced to California in the latter 19th century, but unlike Striped Bass, it is only within the past 30 years they have become an abundant species in the Delta (Conrad et al., 2016). Largemouth Bass is a relatively stenohaline, freshwater ambush predator with a generally littoral distribution not extending substantially seaward of the Delta.

## 3 METHODS

### 3.1 Question 1: At what water temperature threshold will salmon not survive transit through the Delta, and how does that compare to indicators of predation risk?

Kjelson and Brandes (1989) showed that during salmon survival experiments in the 1980s, survival was nearly zero once water temperatures in the Sacramento River reached 20°C. We reevaluated Kjelson and Brandes’ (1989) results using telemetry data from acoustic‐tagged Chinook Salmon smolts that had been released in the Sacramento River basin between 2012 and 2019. We included groups for which 40 or more smolts were known to have entered the Delta (defined by being detected at Freeport, CA, or anywhere downstream). This resulted in data for 19 release groups, consisting of multiple runs: wild‐origin spring‐run (Cordoleani et al., 2018), hatchery‐origin spring‐run (Singer et al., 2020), and hatchery‐origin fall run (Singer et al., 2020; Zeug et al., 2020). Acoustic tags were detected at locations consisting of sufficient individual listening devices deployed in a row to allow for cross‐section coverage of a waterway. Through‐Delta survival was estimated for each release group, and our estimates represent survival from Freeport to the Benicia Bridge detection location (Benicia, CA), a span of 99 river km. Because tagged fish can be missed at individual detection locations, survival and detection probability were both estimated using a Cormack‐Jolly‐Seber model for live recaptures, modeled to vary by detection location and release group. To appropriately estimate detection probability at Benicia, 3–4 additional detection locations downstream were included in the survival model. These included two locations that were always available in proximity to the Golden Gate Bridge, and in certain years a secondary location at Benicia Bridge, west of the location mentioned above. Water temperature experienced by each smolt release group was estimated by calculating the mean water temperature at the USGS Sacramento River gauge below Georgiana Slough (USGS gauge 11,447,903) for a period starting at the mean fish arrival time at Freeport (the northern edge of the Delta) and ending 72 hr later. This 3‐day period was meant to approximate the travel time of most smolts between Freeport and Benicia. We summarized the survival data graphically with overlays of the water temperature effect in a recently derived index of the landscape‐scale predation risk faced by Chinook Salmon smolts (Michel, Henderson, et al., 2020) and information on burst swim speed impairment at elevated water temperature (Lehman et al., 2017). We did not statistically analyze this relationship due to its covariance with river flow and water turbidity as described by Kjelson and Brandes (1989). However, the confidence intervals on the telemetry‐based estimates of survival allowed for objective evaluation of whether fish were likely to survive passage through the Delta at water temperatures near 20°C.

Next, we evaluated whether predator foraging conditions were consistent with an MPE. To do this, we investigated small‐scale spatial distribution and temperature‐related trends in encounter and attack rates of Striped Bass and Largemouth Bass. Information on predator encounters and attacks was compiled from video footage of predation on salmon smolts tethered to floating Predation Event Recorders (PERs; Demetras et al., 2016; Michel, Smith, et al., 2020). The identity of the attacking predator was determined in 222 videos; 197 by Striped Bass, 16 by Largemouth Bass, and 9 by other species. The location of each PER was recorded by a GPS logger, allowing us to calculate the distance (m) to the nearest riverbank when each attack occurred, as well as during times when no predation occurred.

We hypothesized the PER data could demonstrate an MPE if the fundamental mechanism was related to temperature‐dependent changes in predator hunting strategies. If so, we expected to see increases in encounter/attack rates of both Striped Bass and Largemouth Bass at higher water temperature. However, tethered smolts cannot behave freely. Therefore, if the fundamental mechanism for an MPE is related to salmon behavior (i.e., decisions about microhabitat use while emigrating, foraging, or resting), an MPE may not be detectable using PERs.

To evaluate statistical support for the MPE hypothesis, we compiled two datasets. The first included all verified Largemouth bass predation events, along with all PER deployments with no predation. The second dataset included all verified Striped Bass predation events, along with all PER deployments with no predation. Each dataset was assigned a categorical variable of whether water temperatures were above or below 20°C, as recorded from deployed water temperature loggers. We divided the data into two temperature bins because 20°C represented the approximate midpoint of the river temperatures during the study resulting in approximately equal sample size in each bin (mean = 19.9°C, median = 20.4°C, range = 15.2–24.2°C). Cox proportional hazards models were then generated for each dataset to test the null hypotheses that there were no effects of PER distance from shore or categorical water temperature on the likelihood of attack on salmon smolts. Models were generated using the “survival” package in the R environment (R Core Team, 2018). We started with a null model (hourly survival ~1) and then compared it with a model including distance from shore (m) as the only predictor variable. Next, we tested the effect of temperature (less than or greater than/equal to 20°C). In the third iteration, we tested for a combined additive effect of distance from shore and temperature. Finally, we tested the interaction of distance from shore and temperature. We compared the AIC of the four model variations to determine which was the best‐supported and report the p‐value of the best‐fitting model. We also plotted the predictions from the best‐fitting model.

### 3.2 Question 2: Can we recreate empirical survival estimates with simulation modeling?

We developed three predation models to explore our second study question. For the first model, we estimated daily per capita prey consumption by Striped Bass and Largemouth Bass as a function of water temperature using the prey consumption equations from bioenergetics models (Hartman & Brandt, 1995; Rice et al., 1983). For simplicity, we did not use the metabolic calculations that predict predator growth because predator growth over salmon travel time scales would not affect the calculations enough to necessitate the increased complexity. We calibrated the results to our study system using weights of 430 and 450 g for Striped Bass and Largemouth Bass, respectively (Michel, Smith, et al., 2020), and assuming these fish feed at 60% of maximum consumption rates (Nobriga et al., 2013). We plotted the results to show how each predator's fundamental metabolism responds to warming water temperature.

Our second model simulated smolt survival using two different travel time assumptions to explore estimated predation impacts by Striped Bass (scenario 1), Striped Bass + Largemouth Bass (scenario 2), and both predators + an escape ratio *E*, based on decline in Chinook Salmon swimming speed (scenario 3; Table 1; Lehman et al., 2017). Conceptually, *E* either implicitly increases the per capita predation rate on smolts without specifying the predator or increases mortality from a physiological mechanism like disease. We implemented *E* as a function of water temperature: ≤18°C, *E* = 1; at 20°C, *E* = 0.94, and at 22–24°C, *E* = 0.60.

**TABLE 1:** Bioenergetics‐based simulation model scenarios used to predict predation mortality on Chinook Salmon smolts in California's Sacramento‐San Joaquin Delta (USA)

Scenario	*N_SB_*	*N_LB_*	Escape (*E*)
1	677	0	No
2	677	677	No
3	677	677	Yes

Note

The numbers of Striped Bass and Largemouth Bass (*N_SB_* and *N_LB_*, respectively) are per 1,000 salmon smolts. For scenario 1, *n* = 677 Striped Bass per 1,000 smolts produces a calibrated smolt survival target of 0.800 for a 3.47 d travel time at 11°C. The escape term *E* in scenario 3 increases daily mortality based on declining swim speed at high temperature. Each scenario was repeated using smolt travel times through the Delta based on acoustic‐tagged individuals and coded wire‐tagged individuals. On average, the latter take longer to migrate through the Delta.

For each scenario, we generated *n* = 2,000 binomial survival estimates (*S*) at seven water temperatures ranging from 12°C to 24°C in 2°C increments:

(1) $$S={\left[\left(1‐M\right)E\right]}^{x},$$

where *M* is daily mortality, *x* is travel time through the Delta in days, and *E* was defined above. We implemented *x* probabilistically using random draws from lognormal distributions. Parameters for the lognormal distributions were determined using the MASS package in the R environment (R Core Team, 2018). Each of the three scenarios was separately simulated using travel time information informed by acoustic tags and coded wire tags because acoustically tagged fish emigrated through the Delta faster than coded wire‐tagged fish; mean travel times were 3.47 and 9.55 d, respectively. One probable reason is that smolts must be larger to support the increased body burden of an acoustic tag (Figure 1a). Larger smolts are likely more ready to transition to sea than smaller individuals that may still be undergoing some physiological changes needed to support life in the marine environment. Per Equation 1, predation is a time‐dependent process so we were interested in quantifying the different cumulative effects predicted by each data source.

Equation 1 applies the same mortality rate for salmon every day they travel through the Delta. This is unlikely to be realistic (Michel, Henderson, et al., 2020; Michel, Smith, et al., 2020), but beyond water temperature, we did not have data on daily timescale drivers of change in predation rate or its possible links to salmon density. Mortality (*M*) in equation 1 was modeled as:

(2) $$M=[\left(N_{\mathrm{SB}}C_{\mathrm{SB}}\right)+\left(N_{\mathrm{LB}}C_{\mathrm{LB}}\right)]/1000,$$

where *N_SB_* and *N_LB_* are numbers of Striped Bass and Largemouth Bass, respectively (Table 1). The number of Striped Bass used was calibrated to generate a smolt survival of 0.800 (80.0%, or a 20% predation loss) for a 3.47 d migration through the Delta at a temperature of 11°C. This calibrated the model to an approximate upper limit of survival for a tagged salmon release group (Buchanan et al., 2018). At higher water temperatures, model variations can diverge due to differences in underlying assumptions among scenarios.

In Equation 2, *C_SB_* and *C_LB_* are the per capita estimates of the number of Chinook Salmon smolts consumed by each predator based on the bioenergetics modeling described above. Using Striped Bass as the example, *C_SB_* was estimated as:

(3) $$C_{\mathrm{SB}}=\left(B_{\mathrm{SB}}F_{\mathrm{SB}}\right)/7.8,$$

where *B_SB_* is the daily prey biomass in grams estimated to be consumed by Striped Bass, and *F_SB_* is the fraction of consumed prey biomass comprised of salmon smolts. In Michel, Smith, et al. (2020), 7.8 g was the estimated mean weight of a salmon smolt converted from a mean length of 83.3 mm using the length‐weight equation provided by Kimmerer et al. (2005). Based on retrieval of DNA from predator stomach content, Michel et al. (2018) observed *F_SB_* and *F_LB_* to average 0.048 and 0.028 for Striped Bass and Largemouth Bass, respectively. We implemented *F_SB_* and *F_LB_* probabilistically using “rlnorm” in the R environment (R Core Team, 2018) with a bias correction for Jensen's inequality that ensured large samples would return the desired means on the natural scale. The choice of standard deviation for the random draws was arbitrary but mimicked high spatial variability in predation risk (Michel, Henderson, et al., 2020; Michel, Smith, et al., 2020). Evaluation of histograms (not shown) indicated individual random draws could predict *F_SB_* and *F_LB_* up to about 0.2.

Since our approach generated *n* = 2,000 estimates of *S* for each water temperature in each scenario, we consolidated each unique set of estimates into 20 groups of 100 predictions, took the average of each group, and summarized the group‐averaged results using boxplots to avoid plotting results that depict only 0 or 1. The boxplots enabled us to determine whether results from individual scenarios objectively differed from one another.

Our third simulation model used the PER results in place of a bioenergetics‐based approach to predict predation mortality in a region of high predation risk (Michel, Henderson, et al., 2020; Michel, Smith, et al., 2020). Based on the PER and bioenergetics results, we limited this third simulation to Striped Bass. The hourly survival of tethered salmon estimated using PERs is likely to be an underestimate because the smolts have limited ability to escape an attack (Figure 1b). Therefore, we assumed that surviving fish had not been attacked such that 1–surviving fish represented the hourly attack rate, *A_hr_*. Not all Striped Bass attacks on free‐swimming prey fish are successful so we used the linear regression equation provided by Hartman (2000) to predict the fraction of attacks that would result in successful capture (*CAP*) of an untethered smolt: *CAP* = 0.861 – (1.82*PPR). Here, *PPR* is the prey to predator length ratio derived from a laboratory experiment in which Striped Bass were fed cyprinid fishes (*Notropis* spp.) of variable length. As in the second simulation model, we estimated a daily survival of salmon smolts *S*:

(4) $$S={\left[{\left(1‐\left(A_{\text{hr}}*\text{CAP}\right)\right)}^{24}\right]}^{x}$$

where *A_hr_* and *CAP* were defined as above, the exponent 24 converts the hourly estimates into daily estimates, and *x* is travel time in days. We implemented *A_hr_* and *CAP* probabilistically using “rnorm” in the R environment (R Core Team, 2018). We generated *n* = 2,000 random salmon lengths assuming a mean of 83.3 mm and a standard deviation of 8.4 mm (Michel, Smith, et al., 2020) and an associated *n* = 2,000 Striped Bass lengths assuming a mean of 236.5 mm and a standard deviation of 16.87 mm (Nobriga et al., 2013). We generated predictions of mean survival (*S*) and histograms of variability in the predictions for *x* = 3.47 d and *x* = 9.55 d, the mean travel times for acoustic‐tagged and coded wire‐tagged smolts, respectively. The R code for all three simulation models is available in the archived data (https://doi.org/10.5061/dryad.h70rxwdjn).

### 3.3 Question 3: What are the implications for smolt survival of river flow and water temperature relationships in the Delta?

Water temperature affects landscape‐scale predation risk experienced by Chinook Salmon smolts (Michel, Henderson, et al., 2020). Most of the Delta has wide channels and limited numbers of tall riparian trees due to extensive channelization, levee armoring, and road‐building. Further, tidal flows dominate water movement so the temperature of inflowing river water rapidly equilibrates with ambient air temperature, limiting the role that freshwater inflows have on water temperature to relatively short timescales (Wagner et al., 2011). However, short time scales are relevant to the time scale of Chinook Salmon smolt migration through this channelized ecosystem.

We used river inflow data from the California Department of Water Resources’ DAYFLOW database (https://data.cnra.ca.gov/dataset/dayflow) and water temperature grab samples from California Department of Fish and Wildlife fish surveys (https://wildlife.ca.gov/Regions/3) to evaluate the relationships between monthly mean river inflow and monthly mean water temperature along primary salmon migration routes through the Delta. The sampling stations used to characterize water temperature were picked relative to their proximity to each inflow source (https://doi.org/10.5061/dryad.h70rxwdjn). We used inflow and temperature data for April‐June 1995–2019, recognizing that relationships would change each month as weather warms during spring. Note that most of the time frame we examined represents a period of greater environmental regulation that has coincided with what has recently begun to be considered a “megadrought” in California (https://www.ppic.org/blog/californias‐21st‐century‐megadrought/).

We tested the null hypothesis that monthly mean inflow does not affect monthly mean water temperature along primary migration routes through the Delta using an information‐theoretic approach. We used the glm function (with an identity link) in the R environment (R Core Team, 2018) to test a series of models of increasing complexity. A separate series of models was developed for Sacramento and San Joaquin River inflows. We started with a null model (temperature ~1), then compared it with a model including river inflow as the only predictor variable. Next, we added month (April, May, or June) to the model. Finally, we added an inflow by month interaction term. We compared the AIC of the four model variations to determine which was the best‐supported and report model fit as percentage of the null deviance explained by each model. We also evaluated the relationships graphically with scatterplots informed by loess smooths. This qualitative analysis helped inform us which basin by month combinations appear to diverge from a linear relationship.

## 4 RESULTS

### 4.1 Question 1: At what water temperature threshold will salmon not survive transit through the Delta, and how does that compare to indicators of predation risk?

**FIGURE 3:**
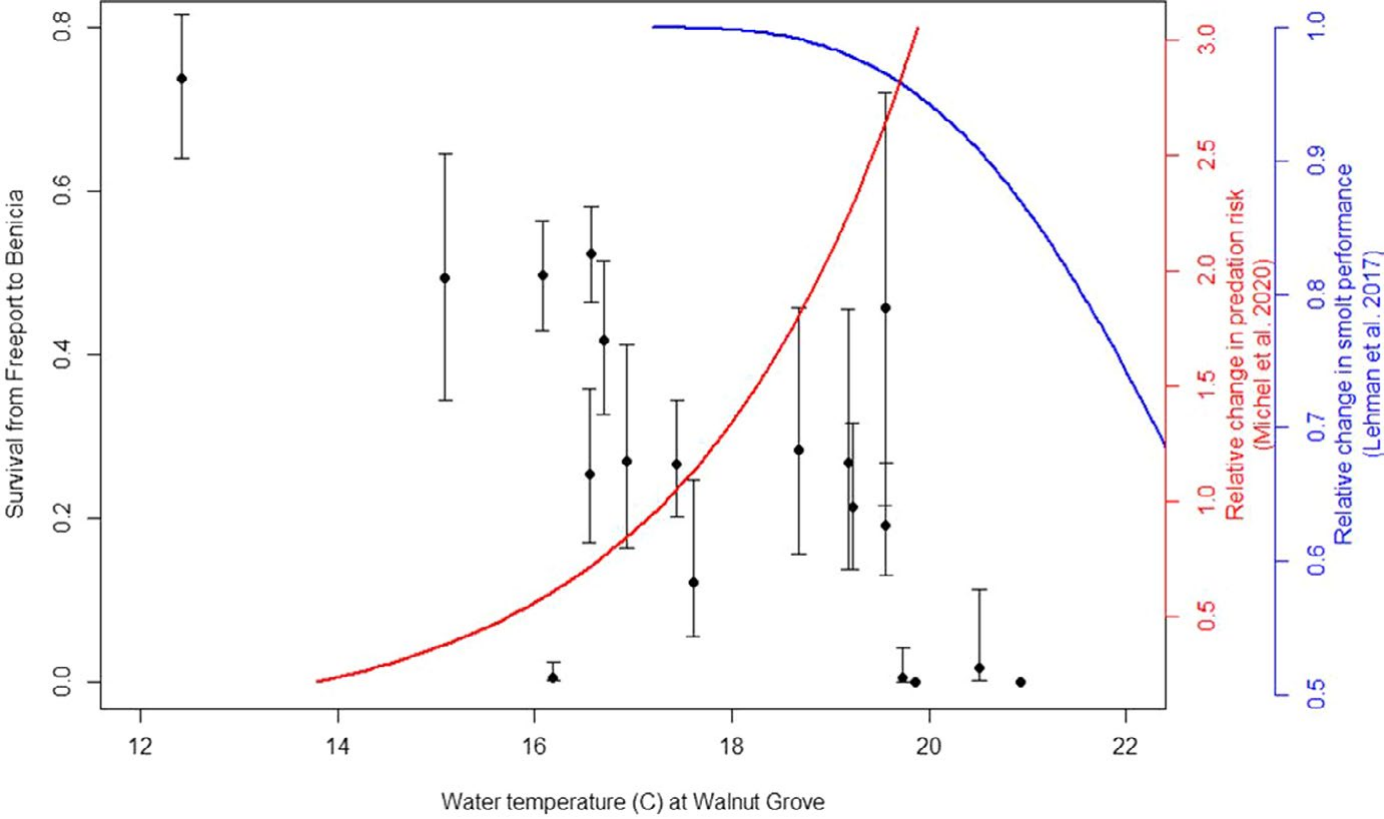
Relationship between water temperature at the Sacramento River below Georgiana Slough gauge (USGS 11,447,903), and the survival of Chinook Salmon smolts defined as successful emigration from Freeport to Benicia Bridge. Point estimates of survival (and 95% confidence intervals) are for individual release groups with at least 40 individuals known to have reached Freeport (entry into the Delta) from upstream release sites. Trend lines depicting the temperature dependency of smolt swimming performance derived from Lehman et al. (2017) and predation risk derived from Michel, Henderson, et al. (2020) are also included

Acoustic tag data for the Sacramento River show juvenile Chinook Salmon survival was highest in a single release group exposed to water temperature near 12°C (Figure 3). Survival was highly variable with strongly overlapping confidence intervals at temperatures ranging from about 14.5° to 19.5°C. One release group exposed to water temperature near 16°C had near‐zero survival, but point estimates for thirteen other groups released into these intermediate temperature conditions ranged from about 0.2–0.5. Survival was nearly zero for two smolt release groups exposed to water temperatures closest to 20°C and two others exposed to slightly warmer water. Qualitatively, this abrupt decline in survival coincides with declining swimming capacity and increasing predation risk. This synthesis of recent data based on acoustically tagged smolts reinforces earlier studies that similarly indicated young Chinook Salmon must emigrate through the Delta before water temperature reaches 20°C.

**FIGURE 4:**
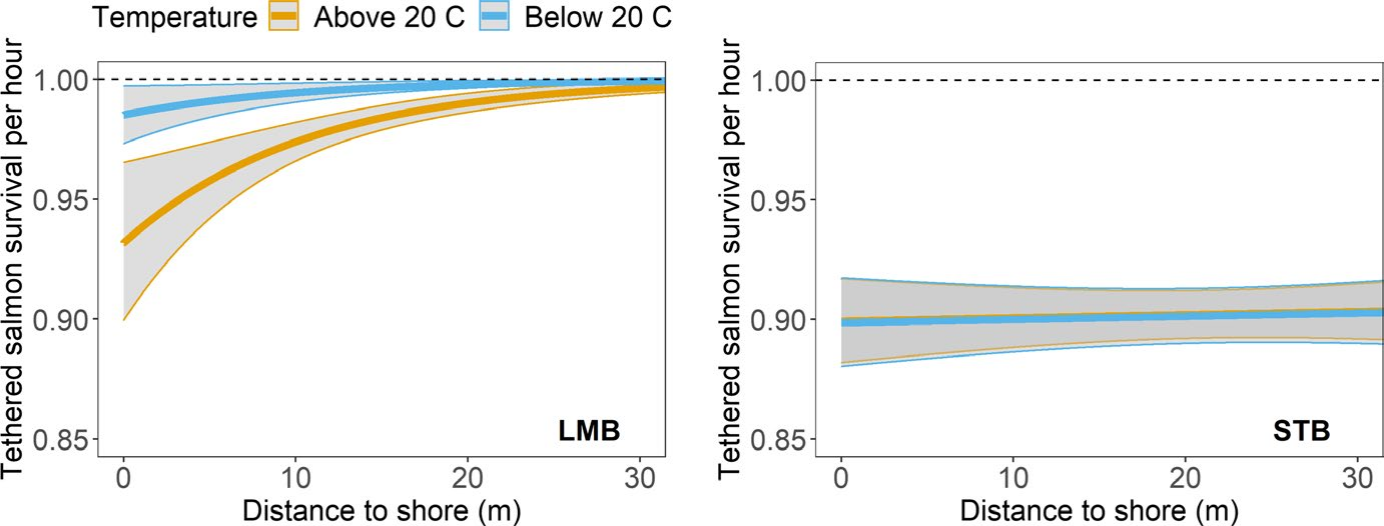
Predicted hourly survival of tethered salmon attached to predation event recorders (PERs) as a function of distance to shore (m, on the x‐axis) and water temperature (above 20°C in orange and below 20°C in blue). Separate predictions were developed for Largemouth Bass (left panel “LMB”) and Striped Bass (right panel “STB”). Predicted survival is generated using the distance + temperature models for both predators (Table 3). Vertical shading represents ±1 SE

Neither distance from shore nor water temperature was observed to influence the willingness of Striped Bass to attack PERs (Table 2; Figure 4). Thus, the null model was the best‐supported hypothesis for Striped Bass as a smolt predator in the Delta. This is consistent with the general depiction of Striped Bass as a temperate pelagic predator (Figure 2). In contrast, Largemouth Bass attacked the PERs most frequently in warmer water, near shorelines (Table 2; Figure 4). This is also consistent with the general depiction of Largemouth Bass as a littoral warm water predator (Figure 2). Considering the two predators together, the PER results support an additive two predator effect because smolts faced a higher nearshore predation risk in warm water. As detailed in the Discussion, we could not effectively support or refute the occurrence of an MPE using these data. We conclude the combined predatory impact of Striped Bass and Largemouth Bass appears additive because there was no observed change in encounter/attacks by Striped Bass when and where Largemouth Bass attacks increased (Figure 4). These data explicitly capture predator activity but not changes in smolt behavior or susceptibility. Additional research is required to properly characterize the extent to which the two predator model results in an additive or synergistic impact on smolt survival.

**TABLE 2:** Results of Cox proportional hazard model analysis of the Predation Event Recorder (PER) data in California's Sacramento‐San Joaquin Delta based on Akaike information criterion (AIC)

Model description	Striped bass	Largemouth bass
	AIC	AIC
Null model (response ~1)	**2,650**	217
Response ~distance from shore	2,652	207
Response ~temperature (binned)	2,652	213
Response ~distance + temperature	2,654	**204**
Response ~distance*temperature	2,655	205

Note

The models predict survival of tethered Chinook Salmon smolts as functions of distance to shore (m) and water temperatures greater or less than 20°C. Separate models were generated using verified Largemouth Bass predation events and verified Striped Bass predation events. The best model for each predator is bolded. The null model indicates no statistically significant influence of distance from shore or water temperature on predation risk. In the best Largemouth Bass model, parameters had *p* ≤ .041. In models of the same data set where the AIC of a higher parameter model is at least 2–7 units lower than a simpler model, the added model complexity is considered to be statistically supportable.

The bolded values represent the lowest AIC and reflect the best supported model variation.

### 4.2 Question 2: Can we recreate empirical survival estimates with simulation modeling?

**FIGURE 5:**
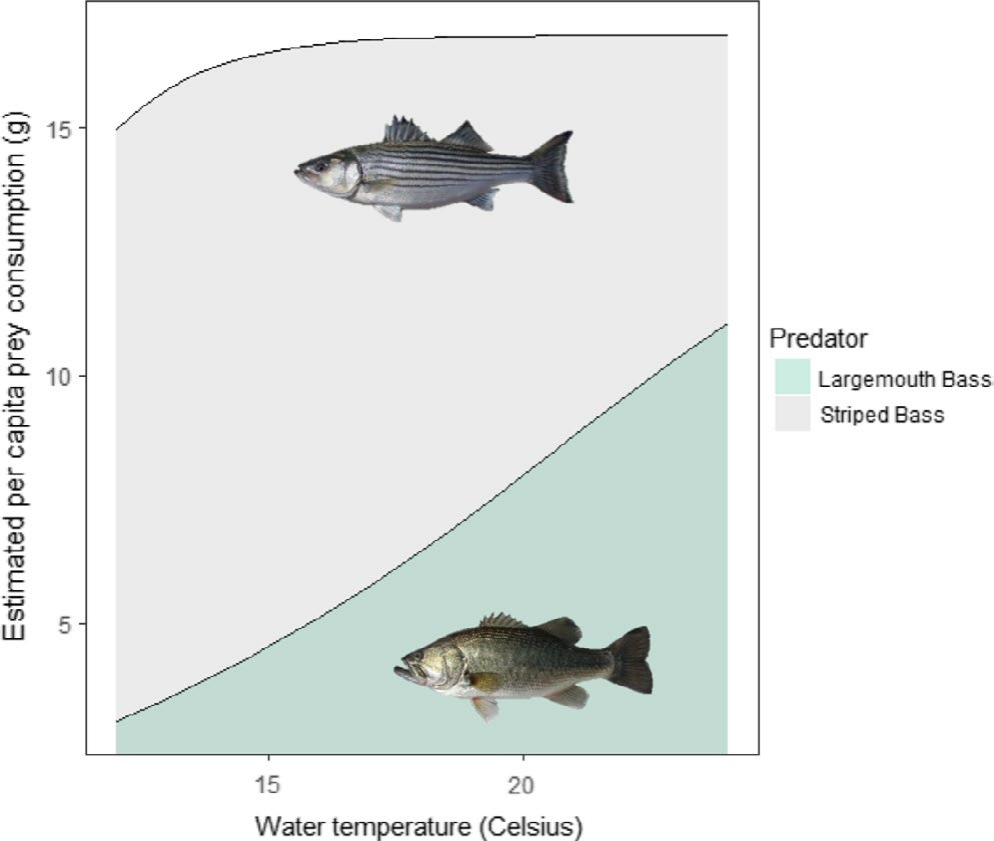
Estimated daily prey consumption by a 430 g Striped Bass (beige) and a 450 g Largemouth Bass (blue‐gray) as functions of water temperature assuming each predator is feeding at 60% of its maximum rate of consumption. Each predator is pictured within its own energetics curve. Photo credits belong to Rene Reyes (https://www.usbr.gov/mp/TFFIP/photo‐gallery‐fish‐south‐delta.html)

The bioenergetics modeling of Striped Bass and Largemouth Bass reinforces the PER results. Striped Bass prey consumption is predicted to hover around 17 g d^−1^ over most of our modeled temperature range (Figure 5). Thus it is not surprising that Striped Bass did not attack PERs more frequently in warm water (Figure 4). In contrast, Largemouth Bass prey consumption is predicted to increase several‐fold over the same temperature range, increasing from about 2–11 g d^−1^ (Figure 5), which is also consistent with what was observed using PERs (Figure 4).

**FIGURE 6:**
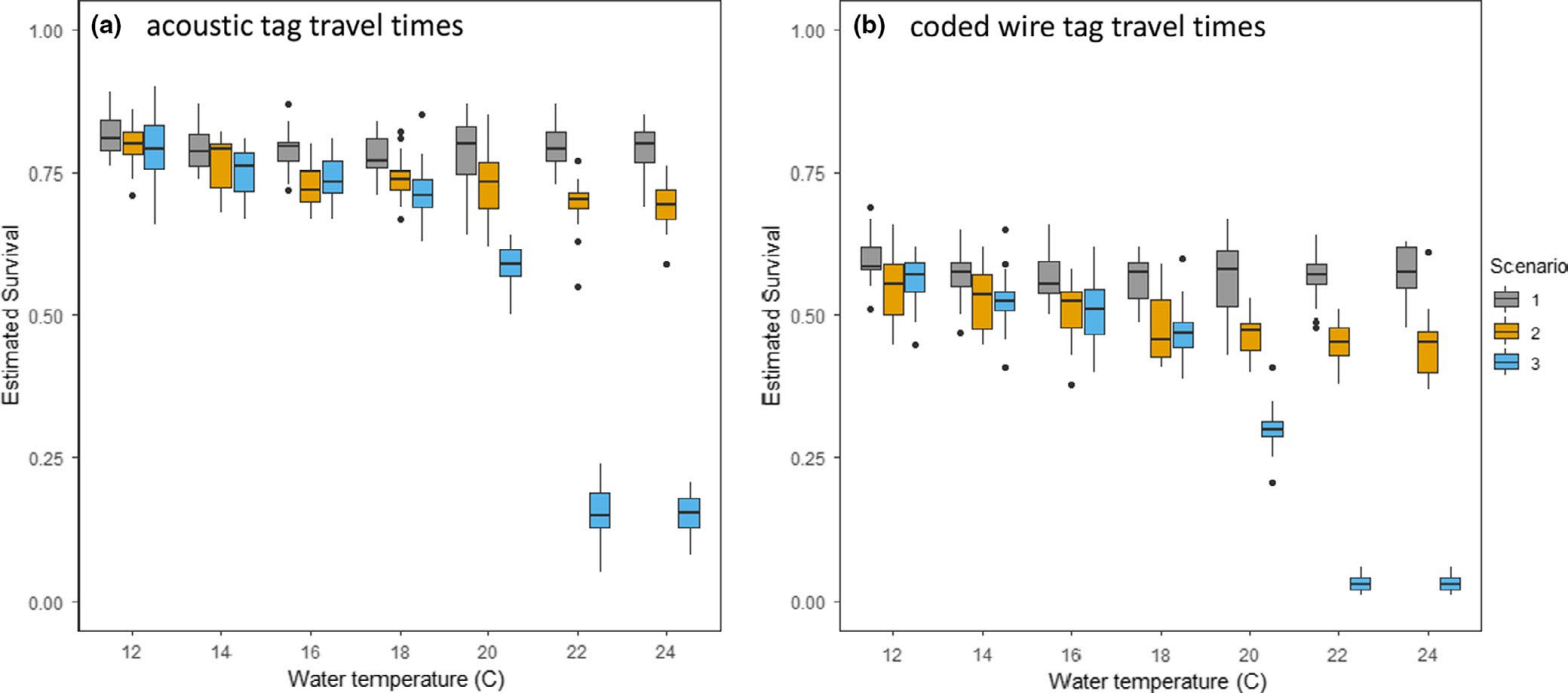
Results of predicted Chinook Salmon smolt survival from bioenergetics‐based simulation model scenarios using travel times of acoustic‐tagged individuals (panel a) and coded wire‐tagged individuals (panel b). Variation depicted in the boxplots was driven by individual variation in *n* = 2,000 smolt travel times and variation in predator diet composition. Predicted survival is lower in panel B due to longer average travel times of coded wire‐tagged fish. Otherwise, scenarios are the same for each panel. In scenario 1, Striped Bass is the only predator. Scenario 2 doubles the number of total predators by adding an equal number of Largemouth Bass. Scenario 3 adds additional daily mortality at water temperatures warmer than 18°C. See Table 1 for scenario input details

In our bioenergetics‐based salmon survival simulation model, we calibrated Striped Bass predation to result in a mortality of 0.2 at 11°C when smolts emigrate the Delta in 3.47 d. Because Striped Bass metabolism is not responsive to the modeled water temperature range, simulated salmon survival in acoustic tag scenario 1 remained near the calibrated value of 0.8 (i.e., 1–0.2) over the entire modeled temperature range (Figure 6a). Longer average travel times using coded wire tag data lowered scenario 1 survival predictions to a median of 0.57 (Figure 6b). The addition of Largemouth Bass in scenario 2 did not have a large impact on predicted salmon survival and introduced only a slight water temperature trend (Figure 6a,b). In scenario 2, median smolt survival was predicted to decline by at most 0.10–0.12 as temperature increased from 12° to 24°C, but the probabilistic implementation caused substantial overlap at every temperature. The implication is that spatial variation in predator diet composition and individual differences in smolt travel times might be able to mask the temperature dependence of Largemouth Bass predation. The addition of the swim speed term *E* had a large quantitative effect on predicted smolt survival, particularly at the two highest water temperatures (Figure 6a,b; scenario 3). However, even the scenario 3 survival predictions were well above zero at 20°C.

**FIGURE 7:**
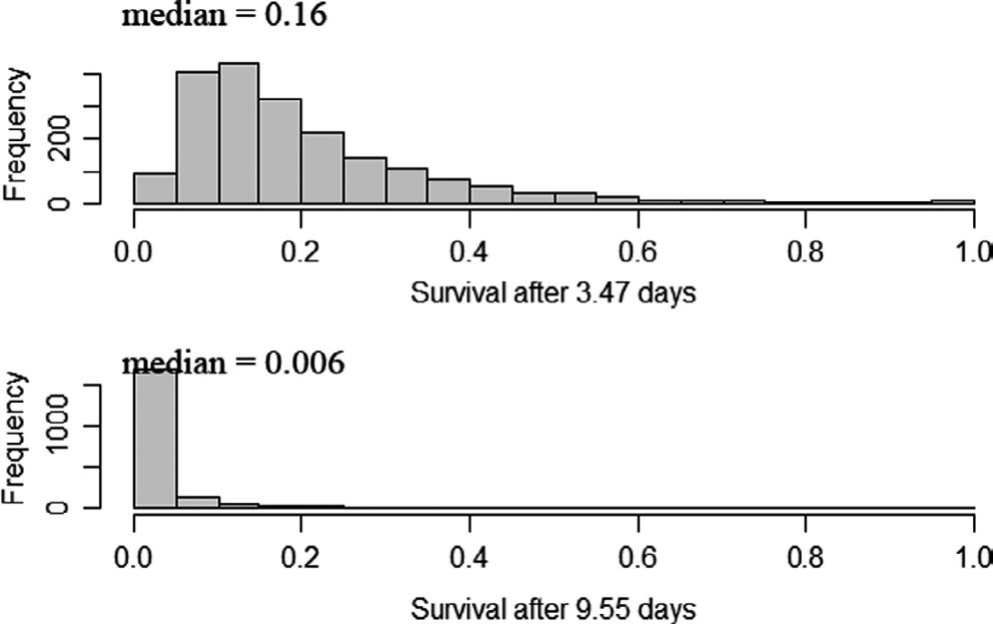
Histograms of *n* = 2,000 Chinook Salmon smolt survival predictions under two assumptions of travel time through California's Sacramento‐San Joaquin Delta. Predictions represent fractions of smolts expected to survive predation by Striped Bass based on results of a predator removal experiment in a known region of high salmon mortality in the San Joaquin River (Michel, Henderson, et al., 2020; Michel, Smith, et al., 2020). In the simulations, variation reflects individual differences in smolt travel time and the ratio of smolt length to Striped Bass length

Our third simulation (Figure 7) was based only on Striped Bass PER results and predicted lower smolt survival than the scenario 2 bioenergetics‐based simulations that included both predators. For a 3.47 d travel time, predation by Striped Bass was predicted to result in a median survival of 0.16, though individual predictions varied widely reflecting the random sizes of prey and predator that “encountered” one another in the simulation. For a 9.55 d travel time through the Delta, variability in predictions was much lower and the median prediction declined to 0.006.

### 4.3 Question 3: What are the implications for smolt survival of river flow and water temperature relationships in the Delta?

**FIGURE 8:**
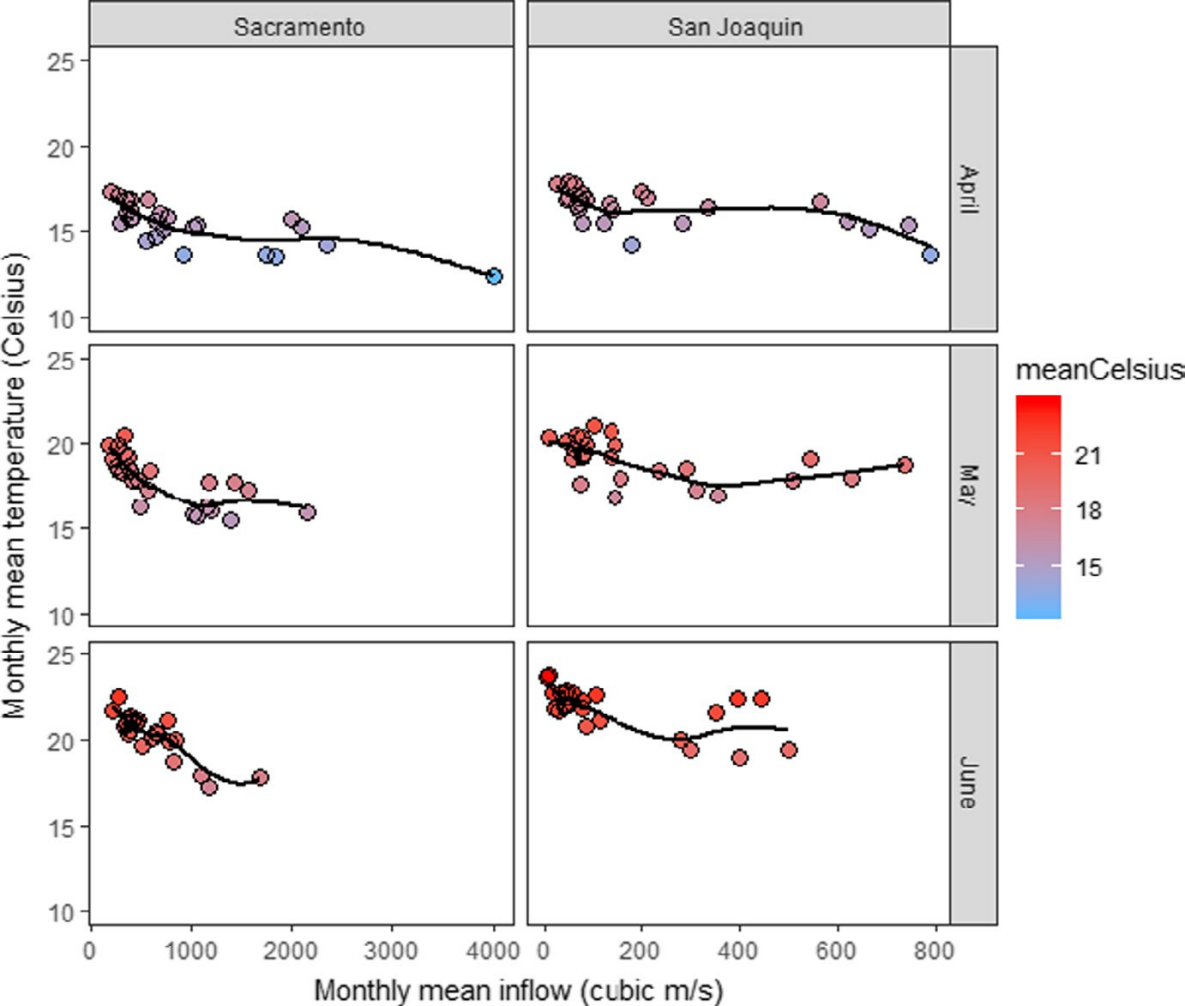
Scatterplots and loess smooths showing the variable, but generally inverse relationships between monthly mean inflow to California's Sacramento‐San Joaquin Delta and water temperature. The color scale in the legend reflects monthly mean water temperature within and across figure panels. Inflow data are from the Sacramento River at Freeport (SAC) + Yolo Bypass (YOLO) and the San Joaquin River at Vernalis (SJR) for the months of April, May, and June, 1995–2019; https://data.ca.gov/dataset/dayflow. Water temperature data were averaged from grab samples collected during California Department of Fish and Wildlife's 20‐mm Survey (starting in 1995) and Spring Kodiak Trawl Survey (starting in 2002). The databases housing the water temperature information are available at https://wildlife.ca.gov/Regions/3

During April‐June, there were statistically significant inverse relationships between monthly mean river inflow and monthly mean water temperature in the Delta along associated migratory corridors (Table 3). For the Sacramento River, the best model included the interaction of inflow and month, but for the San Joaquin River, inclusion of the interaction term was not supported by AIC. These models explained 88% and 85% of the respective null deviance in the data. Though model fit was quite good, loess smooths indicated more complexity in some of the monthly relationships (Figure 8). Monthly mean water temperatures ≥20°C have not to date been observed in April, but have been recorded along both migration routes in May and June.

**TABLE 3:** Results of general linear model analysis of the null hypothesis that river inflow does not affect water temperature in California's Sacramento‐San Joaquin Delta at a monthly average time step during the spring outmigration of juvenile Chinook Salmon

Model description	AIC	%Null deviance	AIC	%Null deviance
Null model (response ~1)	348	0	355	0
Response ~inflow	319	33	344	16
Response ~inflow + month	210	85	**215**	**85**
Response ~inflow + month + inflow*month	**193**	**88**	215	86

Note

The best model for each river is in bold text; all parameters in the best‐fitting model for each river had *p* ≤ .003. In models of the same data set where the AIC of a higher parameter model is at least 2–7 units lower than a simpler model, the added model complexity is considered to be statistically supportable. Note that all parameter additions resulted in >7 unit declines in AIC except adding an inflow by month interaction term for the San Joaquin River data set. The %Null deviance column reports how much of each model's null deviance was explained by the parameter(s) in the model.

The bolded values represent the lowest AIC and reflect the best supported model variation.

## 5 DISCUSSION

We reaffirmed earlier research (Kjelson & Brandes, 1989) indicating very low Chinook Salmon smolt survival as water temperatures climb toward 20°C each spring in California's Sacramento‐San Joaquin Delta. We also showed that if separated by basin and month, the magnitude of river flow entering the Delta from its two largest tributaries can affect water temperature along primary emigration routes. As such, river inflows exert some influence over the number of days juvenile salmon can safely transit the Delta in a given year, especially when inflows are low (Figure 8). The inflows associated with water temperatures ≤20°C increase from May to June because more water is needed to compensate for higher air temperature. During May and June, reservoirs in the watershed can usually retain all inflowing water without exceeding flood risk curves, so reservoir releases reflect demand for irrigation and compliance with water quality standards rather than natural runoff. At the same time, demand for irrigation water increases throughout the Central Valley due to rising air temperature. These drivers interact to affect how much natural runoff reaches the Delta and water diversions within the Delta further affect how much of the river water reaches the estuary.

We hypothesized that, as water warms, young salmon face an intensifying multiple predator effect (MPE) from pelagic Striped Bass and littoral Largemouth Bass (Figure 2). Further research will be required to confirm or refute a nonadditive impact of the two predators. Our two simulation modeling approaches predicted very different magnitudes of predation and the bioenergetics‐based simulation predicted a smaller quantitative impact of Largemouth Bass predation than we anticipated. Interestingly, our simulations indicate there should be no strong influence of water temperature on predation loss of Chinook Salmon smolts unless it directly coincides with a temperature‐driven decline in their swimming capability. This was an unexpected result and we find it informative in that it can focus future research around three hypotheses. (1) Striped Bass is the primary predator of interest. (2) There is an important but undocumented facilitation role for Largemouth Bass. (3) Disease plays a larger role than predators per se in the temperature dependence of smolt survival. We discuss each of these in more detail below to focus future research into these critical conservation uncertainties.

Hypothesis 1 Striped Bass is the primary predator of interest.

Bioenergetics modeling (Figure 5), PER data (Michel, Smith, et al., 2020; Figure 4), a DNA‐based diet study (Michel et al., 2018), and our simulation models (Figures 6, 7) suggest that predation by Striped Bass has a greater quantitative impact on the survival of Chinook Salmon smolts than predation by Largemouth Bass. However, it is also clear that there is no strong water temperature dependence that will emerge from these data. Striped Bass spawn in the Delta and further upstream on the Sacramento River (Goertler et al., 2021). Striped Bass spawning runs usually start in May when water temperatures in the Delta are often in the 15°C to 20°C range (Figure 8). Striped Bass are not believed to feed while they are spawning, but their reproductive migrations can be rapid (Sabal et al., 2019), and if postspawn adults resume feeding soon after spawning, this would, from a simulation modeling perspective, increase the number and mean size of foraging Striped Bass as temperatures coincidentally rise toward 20°C (Goertler et al., 2021). In turn, more and larger Striped Bass would predict lower smolt survival as a function of water temperature. We do not know how much spawning‐related changes in the demographics of foraging Striped Bass would affect bioenergetics predictions in a simulation model because we cannot model such a scenario objectively using currently available data. Thus, for hypothesis 1, we recommend a study to determine how soon after spawning Striped Bass resume feeding, where resumed feeding occurs, and the fraction of adult Striped Bass that have finished spawning and resumed feeding as a function of water temperature. This information would be essential for objectively evaluating whether predation dominated by Striped Bass can explain the water temperature dependence of Chinook Salmon smolt survival, especially for young salmon migrating down the Sacramento River.

Hypothesis 2 there is an important, but undocumented facilitation role for Largemouth Bass.

The PER results and simulation modeling support an additive version of the conceptual model depicted in Figure 2. Further, based on our bioenergetics simulations, the per capita quantitative impact of predation by Largemouth Bass on salmon smolts appears to be about half that of Striped Bass (Figures 5, 6). In addition to its higher per capita demand for prey, Striped Bass also has a much broader distribution along the estuarine salinity gradient. Thus, it likely has the greater consumptive impact on smolts. However, extreme proliferation of submerged aquatic vegetation (SAV) along the shorelines of the San Joaquin River's shipping channel and its distributaries and flooded islands has reduced the turbidity of these waterways (Hestir et al., 2016) and enabled a substantial increase in Largemouth Bass abundance in the last three decades (Conrad et al., 2016). This multifactor origin for the rise of Largemouth Bass in the Delta is spreading into the Sacramento River (northern Delta) and may represent an example of what a warming climate can be expected to bring to temperate aquatic ecosystems more generally, that is, growing ecological prominence of novel sub‐tropical and tropical species assemblages (Conrad et al., 2016; Hawkins et al., 2020). Further, this suite of ecological changes has coincided with a decline of juvenile San Joaquin River basin Chinook Salmon survival to near zero under most conditions (Buchanan et al., 2018). Given that water diversion from the Delta during the spring has been increasingly regulated since the mid‐1990s, elevated predation resulting from the rise of Largemouth Bass would seem like a logical mechanism to explain declining salmon survival. Simulations derived from PER data suggest predation by Striped Bass in areas known to be predation “hot spots” could result in very low smolt survival at nearly any water temperature if travel times through the region are not rapid (Figure 7). Largemouth Bass could exacerbate low survival, but may do so primarily near the shore in warm water. To fully quantify the combined impact of both predators, robust population estimates of Largemouth Bass are needed as are the harder to quantify seasonal estimates for the highly mobile Striped Bass.

Nonconsumptive predator effects are commonly observed in nature (Peckarsky et al., 2008). We propose a testable revision of how an MPE might work that involves a greater nonconsumptive role for Largemouth Bass. Because Largemouth Bass are generally solitary, territorial, littoral predators, they tend to be continuously distributed over large sections of the Delta. For instance, in three 1‐km reaches of the San Joaquin River, Michel, Smith, et al. (2020) collected an average of 403 Largemouth Bass per reach with a coefficient of variation of only 0.17. This suggests that in parts of the Delta where Largemouth Bass are common, young salmon could be expected to encounter an individual of this predator about every 2.5 m of shoreline, which would represent a nearly constant encounter for rapidly migrating smolts if they were distributed nearshore. In contrast, the schooling Striped Bass tend to have a patchy distribution. Michel, Smith, et al. (2020) collected more than 1,000 Striped Bass in one of their study reaches, but fewer than 100 in the other two. We speculate that juvenile Chinook Salmon exploring nearshore habitats as they migrate toward the ocean would frequently and regularly encounter Largemouth Bass. Regular encounter would likely be perceived as a threat that could coax the young salmon back into the offshore environment where they may encounter Striped Bass less frequently and misgauge the greater threat Striped Bass pose. Thus, for hypothesis 2, we also recommend a study to determine whether Chinook Salmon smolts frequently escape predation by Largemouth Bass by avoiding nearshore habitats that might otherwise serve as a partial refuge from Striped Bass.

Hypothesis 3 disease plays a larger role than predators per se in the temperature dependence of smolt survival.

Our bioenergetics‐based simulation model came closest to matching observations (Figure 3 versus Figure 6) when we included an additional daily survival impact derived from an in situ study of changes in the maximum swimming speed of salmon smolts associated with water quality variation in the San Joaquin River (Lehman et al., 2017). These authors emphasized predation risk as a rationale for their study, and we adopted their rationale by assuming slower swimming speeds translate directly into higher predation mortality. Since water temperatures rising toward 20°C do not appear to be highly stressful to local salmon stocks in captivity (Marine & Cech, 2004), the fundamental cause of the slower swimming speeds in Lehman et al. (2017) could be due either to disease or similar ill effects of contaminant exposure. Disease prevalence in Chinook Salmon stocks from California's Central Valley generally increases as the water temperature of their rearing habitat increases (Lehman et al., 2020) and we concur with these authors that the role of disease in empirical patterns of Chinook Salmon survival warrants further study and focused monitoring.

*Relevance to salmon fry*: We focused on smolts, the actively migrating stage in the juvenile Chinook Salmon life cycle. However, potential for an MPE extends to younger Chinook Salmon fry which rear in the Delta prior to seaward migration and therefore can be exposed to predators for longer durations. Better quantifying the role of predation on salmon fry in the estuary will also be an important future research topic because fry have a more littoral distribution than the actively migrating smolts and may therefore have higher encounter rates with Largemouth Bass (Munsch et al., 2019). Most Chinook Salmon fry enter the Delta during January‐April when water temperatures are quite cool and leave as smolts a few weeks to months later. Most young salmon are leaving the Delta's shorelines and moving down‐estuary in April when water temperatures reach about 15°C (Munsch et al., 2019). These fish are leaving earlier in the spring than they once did (Kjelson & Brandes, 1989) and at a cooler water temperature than what would typically be thought of as physiologically stressful (≥20 Marine & Cech, 2004; Lehman et al., 2017). They may be migrating earlier to escape the intensifying threat posed by multiple predators. We consider this worthy of closer research attention given the shallow‐water habitat limitation salmon fry often face (Sommer et al., 2001) and evidence for strong density‐dependent mortality in natal tributaries under lower flow conditions (Sturrock et al., 2019).

*Management Implications*: The management of water supply in the San Francisco Estuary and its watershed is complex due to increasing human demand which has led to long‐term decline in freshwater for the estuarine environment, (Reis et al., 2019). Moving forward, a warming climate is anticipated to become an increasingly important driver of freshwater supply (Dettinger et al., 2015). Climate projections for the region predict warmer weather, lower spring snowpack in the Sierra Nevada, and as a result, more variable winter runoff and lower spring runoff in Central Valley watersheds that feed the estuary. Warmer weather and lower spring runoff could contribute to lower and warmer inflows similar to what has been observed since 2000 (https://www.ppic.org/blog/californias‐21st‐century‐megadrought/). In the San Joaquin basin where outmigration opportunity is already extremely constrained relative to historical conditions (Sturrock et al., 2019), active intervention may be required to sustain viable populations if conditions cannot be improved. From the perspective of habitat restoration, one cannot disconnect the physical habitat from the need for adequate river flows, but the benefit of each unit of flow can be expected to lessen over time as air temperature warms, a circumstance that can only amplify issues around allocations of limited water resources.

In California's Central Valley, the logical expectation is that climate change will constrict the time available for Chinook Salmon to complete the freshwater phases of their life cycles so restoration actions need to maximize the efficacy of the time they will have. Lone et al. and’s (2017) catch‐22 for roe deer involved two predators, lynx and humans. The roe deer was able to persist because they could avoid the two predators for much of the year and could generally always avoid high exposure risk to both predators simultaneously. Extending this concept to our salmon case study, natural resource managers need to find ways to enable these iconic fish to find and exploit habitats in which predation risk is not excessive. The concept of implementing functional flows (Yarnell et al., 2020) aims to create more seasonally inundated shallow‐water habitats and allow for pulse flows to mimic the recession of the historical spring hydrograph. This could provide salmon with a last chance to transit the river and Delta before waters become exceedingly warm (Michel et al., 2021). Reduction of SAV either through removal or hydraulic modifications could reduce the predation risk posed by Largemouth Bass along the salmon outmigration corridor (Conrad et al., 2016). Upstream of the Delta, restoration offers opportunity to reduce habitat utilized by Largemouth Bass for spawning through isolation or filling of ponded areas, potentially impacting the recruitment dynamics of this predator.

Climate change is affecting predator–prey dynamics, some to the detriment of prey (Peers et al., 2020) and some to the detriment of predators (Barton & Schmitz, 2009). A**s** the global climate continues to warm, changed circumstances can be expected to present challenges when managing species vulnerable to extinction (e.g., Brown et al., 2013) as well as presently more abundant species (e.g., Hazen et al., 2013). Fortunately, generalizable modeling frameworks are emerging that can help applied fish and wildlife managers mechanistically predict context‐dependence in predator–prey systems and how drivers may change along a spatial continuum (Northfield et al., 2017). Such process‐based understanding is essential for effective decision‐making when considering if and how to intervene in naturally and unintentionally changing predator–prey systems.

## CONFLICT OF INTEREST

There is no conflict of interest declared in this article.

## AUTHOR CONTRIBUTION

**Matthew Nobriga:** Conceptualization (equal); Formal analysis (lead); Methodology (equal); Visualization (lead); Writing‐original draft (lead); Writing‐review & editing (lead). **Cyril Michel:** Conceptualization (equal); Data curation (lead); Formal analysis (supporting); Methodology (lead); Visualization (supporting); Writing‐original draft (supporting); Writing‐review & editing (supporting). **Rachel Johnson:** Conceptualization (equal); Methodology (supporting); Writing‐original draft (supporting); Writing‐review & editing (supporting). **John D. Wikert:** Conceptualization (equal); Writing‐original draft (supporting); Writing‐review & editing (supporting).

## Data Availability

The data used in this study are archived with Dryad at https://doi.org/10.5061/dryad.h70rxwdjn. Raw river flow data were downloaded from https://data.cnra.ca.gov/dataset/dayflow. Raw water temperature data were downloaded from https://wildlife.ca.gov/Regions/3. Raw salmon tag data were downloaded from https://coastwatch.pfeg.noaa.gov/erddap/index.html.
